# Calibration of Ring Oscillator-Based Integrated Temperature Sensors for Power Management Systems

**DOI:** 10.3390/s24020440

**Published:** 2024-01-11

**Authors:** Nader El-Zarif, Mostafa Amer, Mohamed Ali, Ahmad Hassan, Aziz Oukaira, Christian Jesus B. Fayomi, Yvon Savaria

**Affiliations:** 1 Department of Electrical Engineering, Polytechnique Montreal, Montreal, QC H3T 1J4, Canada; mostafa.amer@polymtl.ca (M.A.); mohamed.ali@polymtl.ca (M.A.); ahmad.hassan@polymtl.ca (A.H.); yvon.savaria@polymtl.ca (Y.S.); 2 Department of Computer Science and Engineering, University of Quebec in Outaouais, Gatineau, QC J8X 3X7, Canada; aziz.oukaira@uqo.ca; 3 Department of Computer Science, University of Quebec in Montreal, Montreal, QC H3C 3P8, Canada; fayomi.c@uqam.ca

**Keywords:** temperature sensor calibration, DC-DC converter, ring oscillator, system-on-chip (SoC), thermal monitoring, thermal sensor

## Abstract

This paper details the development and validation of a temperature sensing methodology using an un-trimmed oscillator-based integrated sensor implemented in the 0.18- μ m SOI XFAB process, with a focus on thermal monitoring in system-on-chip (SoC) based DC-DC converters. Our study identifies a quadratic relationship between the oscillator output frequency and temperature, which forms the basis of our proposed calibration mechanism. This mechanism aims at mitigating process variation effects, enabling accurate temperature-to-frequency mapping. Our research proposes and characterizes several trimming-free calibration techniques, covering a spectrum from zero to thirty-one frequency-temperature measurement points. Notably, the Corrected One-Point calibration method, requiring only a single ambient temperature measurement, emerges as a practical solution that removes the need for a temperature chamber. This method, after adjustment, successfully reduces the maximum error to within ±2.95 °C. Additionally, the Two-Point calibration method demonstrates improved precision with a maximum positive error of +1.56 °C at −15 °C and a maximum negative error of −3.13 °C at +10 °C ( $R^{2}$ value of 0.9958). The Three-Point calibration method performed similarly, yielding an $R^{2}$ value of 0.9956. The findings of this study indicate that competitive results in temperature sensor calibration can be achieved without circuit trimming, offering a viable alternative or a complementary approach to traditional trimming techniques.

## 1. Introduction

Integrated temperature sensors, notably in CMOS technology, have become indispensable in modern electronics for their compact size, low power consumption, and seamless integration with system-on-chip (SoC) designs. These sensors are renowned for their accuracy, sensitivity, and broad operational range, exhibiting a linear response across diverse applications, from consumer electronics to automotive and industrial monitoring systems. Their integration into various electronic devices is essential for efficient thermal management and reliability [1,2,3]. In the context of modern integrated circuits (ICs), temperature monitoring is a critical task. The increasing power dissipation in densely packed circuits poses inherent challenges, necessitating temperature monitoring solutions that are not only accurate and responsive but also energy efficient. This need is acutely felt in applications ranging from wearable devices to complex industrial systems, where maintaining optimal performance and prolonging the lifespan of electronic components is crucial.

Historically, the domain of temperature sensing in CMOS processes heavily relied on bipolar junction transistors (BJTs). Such methods can translate temperature variations into a digital representation with remarkable accuracy over a vast temperature range [4,5]. However, these BJT-based solutions have challenges, the most significant being their relatively high power consumption [5].

In light of this, temperature sensors based on MOSFETs started gaining attention. Such sensors, especially the ones grounded in Proportional to Absolute Temperature (PTAT) and Complementary to Absolute Temperature (CTAT) principles, emerged as energy-efficient alternatives to their BJT counterparts [6]. Yet, they have limitations, particularly concerning thermal sensitivity and the susceptibility to parametric variations of fabrication processes [7,8,9].

Ring oscillators (ROs), predominantly based on CMOS inverters, have emerged as a promising solution for temperature sensing, and are particularly suited for digital platforms like FPGAs and microprocessors. These oscillators, renowned for their versatility, integrate seamlessly into various digital systems, making them ideal for applications that require detailed thermal profiling. Their frequency variation in response to temperature changes and the digital nature of their output are pivotal for on-chip temperature monitoring. This capability is crucial for thermal management in high-density and high-power scenarios, such as embedded systems, FPGAs, CPUs, and power management in portable devices. The widespread application of RO temperature sensors in fields demanding digital integration and compact design underscores their significance in contemporary electronic systems.

However, as with many types of temperature sensors, the performance of ROs critically depends on their calibration. An uncalibrated sensor cannot be used since it is too sensitive to process variations. Calibration ensures that the sensor’s output aligns closely with the actual temperature. While the conventional One- and Two-Point calibration methods were adequate for many PTAT-based CMOS sensors over broader temperature ranges, they displayed substantial non-linear errors when applied to ROs. This underscores the need for advanced calibration strategies for these oscillators, ensuring their reliability and accuracy over the entire operating range [10].

A prevailing calibration method for temperature sensors involves the use of trimming. This technique entails adjusting the behavior of the temperature sensor at a given temperature to yield a predetermined frequency output. Such a strategy has been documented and adopted in various articles, notably in [11,12]. The research reported in [11] outlines a design utilizing a PTAT current source in the TSMC 65 nm technology. The distinctive feature of this technology is the threshold voltage’s linear proportionality to temperature, which follows a negative trajectory. This PTAT current source was essential in biasing a three-stage conventional RO, producing a temperature-dependent frequency signal. The calibration strategy adopted involved trimming at two distinct temperatures, 20 °C and 90 °C, resulting in a temperature accuracy variance of ±3 °C. On the other hand, the method detailed in [12] was oriented explicitly towards diminishing the implications of process variations. This method entailed the deployment of two ROs to detect temperature. The essential point of this approach hinged on extracting the differential frequency from these oscillators at a specified temperature. Their foresight in devising the temperature-insensitive oscillator (TIO) proved beneficial in mitigating temperature variability and rectifying non-linearity, especially at higher temperatures. Upon testing 15 chip prototypes, they discerned a temperature spread of ±5 °C pre-calibration, which subsequently refined to a range between +2.7 °C and −2.9 °C post-calibration.

Table 1 offers a comprehensive survey of ring-oscillator-based temperature sensors, illustrating the breadth of design strategies and calibration techniques explored in this domain. It underscores the range of approaches, from resistor trimming to sophisticated digital control schemes, implemented across various oscillator configurations such as current-controlled, current-starved, and delay line types. Notably, the table reveals that these sensors consistently achieve accuracies of ±3 °C or better. This level of accuracy highlights the robustness of ring oscillators in providing accurate temperature readings, even amidst the inherent challenges posed by process variations. Their versatility across different technology platforms further cements their role as critical tools in precision temperature sensing, capable of adapting to diverse requirements and constraints.

The continuous evolution of the semiconductor industry, characterized by increasingly dense and high-power devices, necessitates proficient temperature sensing mechanisms. As these developments progress, striking an optimal balance between accuracy, energy efficiency, and mitigation of process variations becomes imperative. In this context, temperature sensors play a critical role in monitoring and managing the thermal performance of such devices.

Given the foundational work we reviewed in the field, our study introduces a novel approach using ring-oscillator-based temperature sensors. While conventional methods predominantly rely on trimming techniques—aligning the sensor’s frequency with a reference value at a predetermined temperature—our research proposes a paradigm shift. Our approach diverges from these conventional trimming methods by integrating process and post-measurement modeling. This dual-pronged strategy aims to leverage the inherent attributes of the sensor and refine its output post-fabrication, yielding precise temperature measurements. Our method thus seeks to blend simplicity with precision, offering an effective counter to the challenges faced by conventional calibration techniques.

Central to our contribution is the proposal and characterization of several trimming-free calibration techniques. These techniques demonstrate versatility and adaptability, ranging from zero to thirty-one frequency-temperature measurement points. Notably, our research showcases the viability of a One-Point Calibration method, which can be conducted from a single temperature measurement performed at ambient conditions, thus eliminating the need for a temperature chamber. This finding is significant as it underscores the limitations of uncalibrated ROs, characterized by a complexity-accuracy trade-off, and highlights the potential of our approach in addressing these constraints.

The rest of this paper is organized as follows. Section 2 presents the proposed RO-based temperature sensor (ROTS) and the related novel calibration techniques. The validation of our sensor design and proposed calibration methods based on simulations and experimental measurements are reported in Section 3. Section 4 concludes the paper by summarizing our main findings.

## 2. Ring Oscillator-Based Temperature Sensor: Design, Implementation, and Evaluation Criteria

### 2.1. Design and Implementation

Our work in this paper focuses on finding an effective way to detect and mitigate thermal peaks in systems that undergo intense thermal stress, such as a monolithic DC-DC converter, which our team is considering [21,22]. While our current implementation is software-based in Python version 3.6, the architecture is designed with the flexibility to adapt to hardware platforms, including FPGA interfaces, for real-time applications. Post-layout simulations of that power converter showed that its power transistors can dissipate a total power exceeding 1.6 W in less than 5 ${\mathrm{mm}}^{2}$ of silicon area. This enormous power dissipation can lead to a significant temperature rise, exceeding 100 °C, depending on the package type. This can affect the device characteristics and the performance of all neighboring circuit blocks, possibly deteriorating the overall performance of the systems where they are embedded. An unmitigated temperature rise of the cited magnitude can lead to irreversible chip damage and system failure.

**Figure 1:**
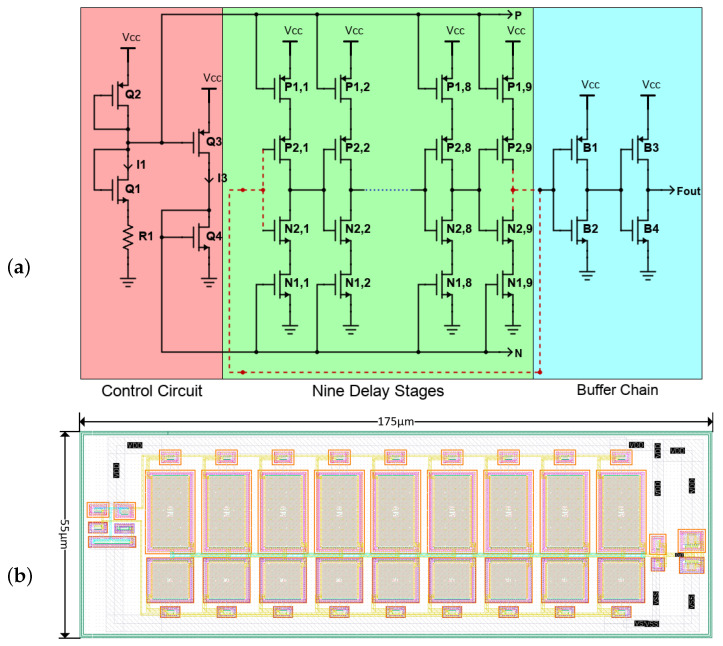
Implementation of the ROTS: (**a**) Transistor-level (top) and (**b**) Layout level (bottom).

To allow accurate and timely detection of thermal peaks, the present paper explores the use of ROTSs. ROs are well known for their ability to monitor on-chip temperature, as their output frequencies vary depending on the temperature of their components. In this paper, we studied and implemented a nine-stage RO composed of three sub-circuits: a control circuit, nine delay stages, and a buffer chain, as shown in Figure 1a.

Inspired by the work presented in [23], the circuit operates as follows: on the left branch, Q1 pairs with R1 to function as a current source, generating the current I1. Transistors Q2 and Q3 form a current mirror, which mirrors the current I3 and I1. Transistors Q2 and Q4 form a separate current mirror connecting the input and delay stages. Consequently, any change in the current I1 affects the signal propagation delay in the delay stages, leading to changes in the output frequency.

The delay stages act as current-starved inverters. Transistors $P_{1,x}$ and $N_{1,x}$ provide the source and sink currents, respectively, through current mirroring with the input stage. $P_{2,x}$ and $N_{2,x}$ act as an RO, for which the oscillation period is [24] is given by (1):

(1) $$T_{osc}=2N\tau \;\mathrm{with}\;\tau =\frac{V_{CC}C_{g}}{I_{CONT}}$$

where $C_{g}$ represents the total parasitic capacitance of the NMOS and PMOS transistors. It comprises two components: the output capacitance of the inverter stage and the input capacitance of the subsequent stage. $V_{cc}$ is the supply voltage and $I_{CONT}$ is the control current.

The buffer stage essentially acts as an inverter designed to increase the circuit’s driving capability and to sharpen the generated signal when connected to an external load. Table 2 summarizes the dimensions of the transistors comprised in the ROTS circuit. Figure 1b presents the sensor’s layout, which spans an area of 175 μ m × 55 μ m when implemented using the XFAB 180 nm technology [25]. In this layout, the central portion comprises the current-starved delay stages. The smaller transistors located on the left represent the control circuit. The buffer chain that implements the core RO is found on the right.

Figure 1a illustrates the temperature sensor circuit. The voltage-current relationship for the left branch of its control circuit is:

(2) $$V_{D1}-V_{thn}=I_{1}\;\cdot \;R1+\sqrt{\frac{2I_{1}}{k_{n}\left(W_{1}/L_{1}\right)}}$$

where $V_{D1}$ denotes the drain voltage of transistor Q1 , $I_{1}$ represents its drain current, and the parameters $V_{thn}$ , $k_{n}$ , $W_{1}$ , and $L_{1}$ correspond to the threshold voltage, transconductance parameter, width, and length of Q1 , respectively.

**Figure 2:**
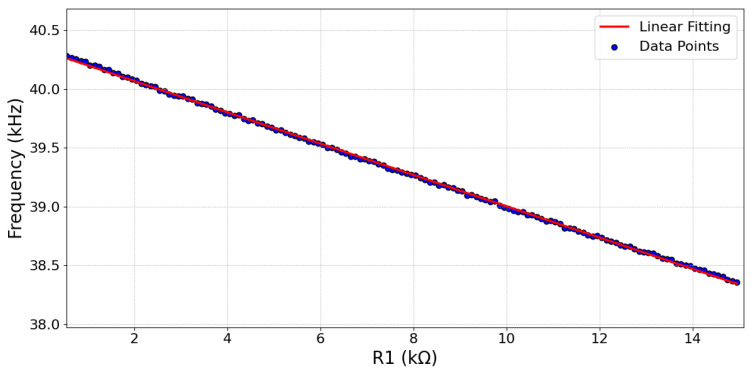
Simulated output frequency of the ROTS as a function of R1 (typical process parameters at 27 °C.

In Figure 1a, the control circuit is identified as the PTAT circuit. This circuit’s resistor is integral in defining the ROTS’s temperature-dependent properties, owing to the resistor’s positive temperature coefficient, denoted as R1 . As temperature increases, the voltage drop across the resistor rises, leading to a decrease in the bias current through Q1 [14]. This change in bias current affects the ROTS output frequency, described by the formula $f=\frac{I_{\mathrm{CONT}}}{2NC_{g}V_{CC}}$ . As a result, there is a reduction in the oscillation frequency with an increase in temperature [24]. The relationship between the resistance value and the output frequency is showcased in Figure 2, illustrating a CTAT relationship through the simulated output frequency as a function of R1 .

In the PTAT circuit under consideration, the resistor plays a crucial role in determining the temperature-dependent characteristics of the ROTS. This behavior is attributed to the positive temperature coefficient of the poly resistor, R1 . As the temperature rises, there is an increased voltage drop across the resistor, subsequently reducing the bias current through Q1 [14]. Such change impacts the ROTS output frequency, given by $f=\frac{I_{\mathrm{CONT}}}{2NC_{g}V_{CC}}$ . This results in a reduction in the oscillation frequency with temperature [24]. Figure 2 depicts the simulated output frequency as a function of R1 , with the CTAT relationship illustrated by blue dots. In the same figure, a linear fit to this data is depicted as a red line. The close alignment of the blue dots and the red linear fit line indicates a near-linear relationship with a negative slope, emphasizing the sensor’s CTAT characteristics.

Our earlier study in [26] demonstrated that a quadratic model more accurately captures the temperature-frequency relationship in a current-starved ROTS. Delving deeper, the resistor in the input stage significantly impacts this relationship. However, the inherent non-linearity arises from the linear shift of the MOSFET threshold voltage with elevated temperature. This, in turn, modulates the MOSFET current based on the Shichman-Hodges model, indicating a direct proportionality to $V_{th}^{2}$ . The ensuing quadratic current behavior inversely affects the delay, as illustrated in (1). Thus, the temperature-delay relationship is best characterized using a quadratic perspective. The efficiency of this approach was substantiated in [26], boasting an excellent coefficient of determination, surpassing 99.7%.

**Figure 3:**
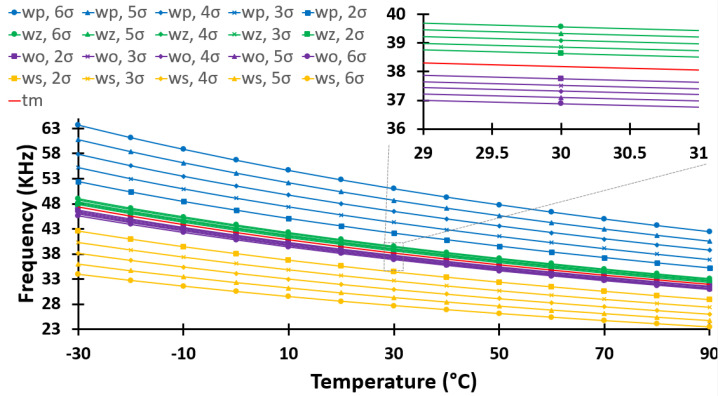
Post-layout simulation performance of the ROTS over process corners: typical (tm), worst-one (wo: fast-n, slow-p), worst-zero (wz: slow-n, fast-p), worst-power (wp: fast-n, fast-p), and worst-speed (ws: slow-n, slow-p), each at 2 σ , 3 σ , 4 σ , 5 σ , and 6 σ .

Figure 3 shows the simulated output frequency of the ROTS as a function of temperature from −30 °C to 90 °C over four process corners: worst-one (wo: fast-n, slow-p), worst-zero (wz: slow-n, fast-p), worst-power (wp), and worst-speed (ws), each at 2 σ , 3 σ , 4 σ , 5 σ , and 6 σ , where σ denotes the standard deviation of the normal distribution of the 180 nm XFAB technology. The reported results show that the frequency generated by the ROTS strongly depends on temperature (as temperature increases, the output frequency decreases).

**Figure 4:**
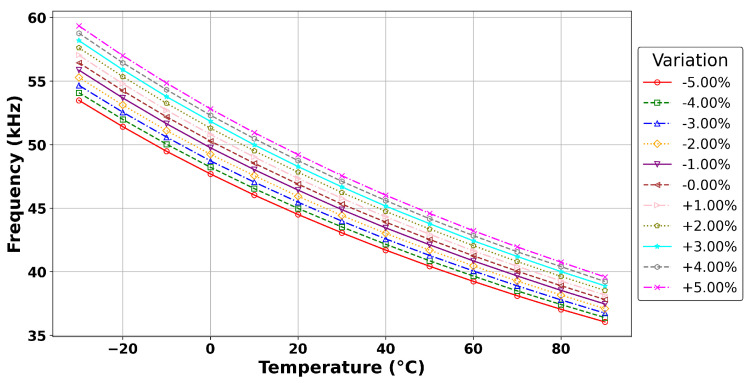
Analysis of the ROTS power supply sensitivity across the typical process corners spanning the −30 °C to 90 °C temperature range.

Figure 4 characterizes the response of the output frequency to power supply voltage variations. Utilizing a baseline power supply of 5 V, we systematically varied the $V_{CC}$ from −5% to +5%. The analysis indicates that as the voltage increases, there is a corresponding increase in current, which subsequently enhances the switching speed and, therefore, the output frequency. Together, these datasets furnish a comprehensive PVT (process, voltage, temperature) profile, providing a foundational basis for employing the interpolation methods proposed in the subsequent section to derive calibrated measurements from an uncalibrated ROTS sensor.

### 2.2. Evaluation Criteria

In our study, we introduce three calibration methods for ring-oscillator-based temperature sensors, with each method specifically developed to address process variations with varying degrees of complexity and accuracy. Alongside these methods, we also consider the Linear Calibration and Second-Degree Polynomial Best Fit Approach as benchmarks. These benchmarks serve to evaluate the effectiveness of our proposed calibration techniques. The One-Point Calibration method simplifies the process by relying on a single measurement point, enhanced by curvature information from corner simulations. The Two-Point Calibration approach integrates two primary calibration points with supplementary data from process corners. The Three-Point Calibration method employs a quadratic model and uses three distinct temperature measurements for a comprehensive system evaluation. The advantage of these calibration-free technologies is significant; they simplify the design and manufacturing process, dramatically reducing costs and time-to-market. Unlike traditional calibration methods, which often necessitate device specific post-manufacturing adjustments, these new methods offer consistency and reliability without individual sensor trimming, making them suitable for large-scale applications.

#### 2.2.1. One-Point Calibration Method

The one-point calibration method simplifies the calibration process by focusing on a single critical point in the measurement spectrum while following the curvature of the closest corners derived from corner simulations. This method assumes that a specific point on the temperature-frequency relationship curve provides sufficient information for accurate device calibration, given its alignment with the curvature of the nearby corners. A primary advantage of this technique is that if the resulting predicted temperature is sufficiently accurate, calibration can be performed based on a single measurement at room temperature.

Although the calibration process is streamlined with just one primary data point, the method employs curvature information from the nearest corner cases to ensure a more accurate calibration. The calibration’s error margin is expected to remain within acceptable limits, even with this reduced dataset. The steps employed in the one-point calibration method include:

- Measure the output frequency at a known temperature T1 (ambient, for instance). This single data point forms the foundation of the calibration.
- Use the corner simulation data to follow the curvature of the closest corners, thereby refining the calibration model based on the device’s specific requirements and characteristics.
- Apply the frequency measurement at T1 and the curvature details from the corner cases to obtain the calibrated response.
- To determine the temperature from the measured frequency, one could utilize the inverse of the established relationship between temperature and frequency. If this relationship is mathematically defined, the inverse function can provide the temperature for a given frequency. Alternatively, a lookup table, populated with calibration data, can be used to find the closest frequency values and interpolate between them to retrieve the corresponding temperature.

Further explanation on the one-point calibration method, considering various scenarios, can be found in our previous work [26].

#### 2.2.2. Two-Point Calibration Method

The Two-Point calibration method arises from balancing efficiency and precision in the calibration process. Reducing the number of calibration points becomes desirable when obtaining multiple ones proves laborious, time-consuming, or resource-intensive. However, to be acceptable, this reduction must not come at the cost of drastically sacrificing the accuracy of the calibration model.

The Two-Point method offers a viable alternative by integrating the data from various process corner cases with the two primary calibration points. This approach effectively leverages the reliability and accessibility of corner case data, treating it as a pseudo-third calibration point. Thus, it provides a foundation for a second-degree polynomial to model the frequency-temperature relationship, even with limited primary data points.

The advantages of this approach are many. Not only does it reduce the overhead associated with data collection, but it also expedites the calibration process. While it acknowledges a tradeoff in absolute accuracy, it ensures that the calibration remains within acceptable error margins. The method also ensures that the reduced calibration model remains robust across a wide range of operating conditions by utilizing process corner case data, often from thorough simulations or extensive device characterization. The specific steps of the Two-Point calibration approach are as follows:

- Measure the output frequency at a known temperature T1 and compare this measurement point with the corner cases from Figure 3. If the measured point does not belong to any curve and lies between two curves, the following weighting equation below is used for interpolation:
(3) $$f\left(T1\right)=\frac{d_{up}f_{dn}\left(T1\right)+d_{dn}f_{up}\left(T1\right)}{d_{up}+d_{dn}}$$
  where $d_{up}$ is the vertical distance (in frequency) between the calibration point and the nearest upper bound curve, $d_{dn}$ is the vertical distance between the calibration point and the nearest lower bound curve, and $f\left(T\right)$ , $f_{dn}\left(T\right)$ , and $f_{up}\left(T\right)$ represent the interpolated Lagrange second degree polynomial of the calibrated data, lower-bound curve, and upper-bound curve, respectively.
- Repeat the same process for a different calibration point at a known temperature T2 to obtain $f\left(T2\right)$ . For higher accuracy, it is advisable to select sufficiently distant calibration points.
- To force the calibrated response curve to pass through the two calibration points while taking advantage of the chip’s corner simulation, we perform the following linear combination:
(4) $$f\left(T\right)=f\left(T1\right)-\frac{\left(f\left(T1\right)-f\left(T2\right)\right)\times \left(T-T1\right)}{\left(T2-T1\right)}$$

#### 2.2.3. Three-Point Calibration Method

The objective of this method is to perform an accurate calibration of the temperature-frequency relationship in a current-starved ROTS. The premise of this method depends on using a second-degree equation, or a quadratic model, to accurately capture the temperature-frequency dependence of the ROTS.

The selection of this quadratic model finds its roots in the observed data patterns, which tend to exhibit a non-linear behavior. This non-linearity stems from the characteristics of the Metal-Oxide-Semiconductor (MOS) transistor, particularly its voltage-dependent threshold voltage. This voltage dependency contributes a significant degree of variability to the data, a variability that a linear model would struggle to capture accurately. Therefore, a quadratic model is inherently more accurate.

However, effective utilization of this approach lies in carefully selecting the calibration points. To enhance the model’s accuracy, it is generally advisable to maintain substantial spacing between the calibration points. The increased spacing ensures that a broader range of data is included in the calibration process, thereby enhancing the model’s predictive capabilities. To implement this Three-Point calibration approach for the ROTS, three distinct measurements must be taken:

1. At a low temperature of $-21^{\;\circ }$ C, capturing the low-end performance.
2. At room temperature, ensuring a central reference point.
3. Finally, at a high temperature of $81.5^{\;\circ }$ C, assessing the high-end performance.

The three distinct proposed measurement points span a broad temperature range, each providing essential data for the calibration process. Using these three data points, a second-degree polynomial is interpolated to represent the performance of the temperature sensor across this temperature spectrum. This method, while simple, leverages the extensive data coverage obtained from the wide-ranging temperatures to calibrate the ROTS effectively. The relationship between temperature and frequency can be characterized with high precision by fitting the observed data to the quadratic model. This ensures the ROTS provides reliable temperature readings across its operating temperature range.

#### 2.2.4. Linear Approach

This method is straightforward, whereby we choose two temperature points, $T_{1}$ and $T_{2}$ , and then obtain their respective measured sensor frequencies, $f_{1}$ and $f_{2}$ . Assuming a linear relationship between temperature and frequency, we interpolate the frequencies for other temperature values based on this assumption.

#### 2.2.5. Second-Degree Polynomial Best Fit Approach

This method operates based on the assumption that the temperature-frequency relationship can be modeled as a quadratic function. After collecting many temperature/frequency measurements, these results are interpolated to obtain a second-degree polynomial. The primary goal of this approach is to minimize the mean square error associated with the sensor’s readings.

## 3. Experimental Setup and Measurement Results

### 3.1. Chip Testbench and Measurements

**Figure 5:**
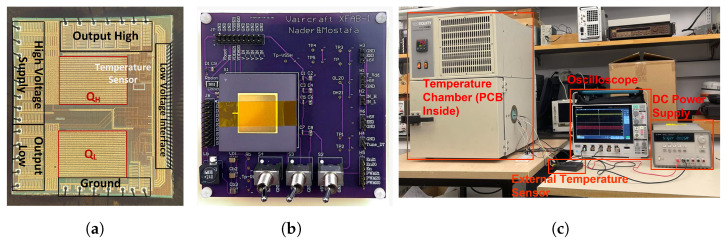
(**a**) Microphotograph of the fabricated XFAB chip prototype detailing the DC-DC converter configuration and the strategic placement of the ROTS near the high-power transistor QH, (**b**) Custom printed circuit board (PCB) designed for chip testing with a user-friendly external interface, and (**c**) Testbench setup employed for the characterization of the fabricated temperature sensor.

Our research team developed a prototype DC-DC converter integrated with an innovative temperature sensor, fabricated using the xfab xt-180 nm CMOS XFAB technology. This sensor is crucial for monitoring the heat generated by the power transistors in the converter, which is a key aspect of ensuring the converter’s efficiency and reliability. The micro-graph of this prototype is depicted in Figure 5a.

To thoroughly evaluate the sensor’s performance, we mounted the chip on a custom-designed printed circuit board (PCB), as illustrated in Figure 5b. This PCB was engineered to facilitate easy interfacing with external test equipment, thus enabling comprehensive testing and analysis.

For the calibration of the sensor, we employed a temperature chamber instead of the DC-DC converter itself. This decision was made as the temperature chamber can accurately simulate the entire operational temperature range of the converter, something the converter alone could not achieve, especially for temperatures below ambient. Furthermore, since the DC-DC converter inherently generates heat, using it for calibration would have limited our ability to test the sensor at lower temperatures. Therefore, the temperature chamber provided a more controlled and versatile environment for calibration, ensuring that our sensor’s readings were accurate across the full spectrum of potential operating conditions.

In our setup, we positioned the test chip with its lid open to directly expose the sensor to the controlled environment of the heat chamber, which was maintained in its standard operational state for precise temperature regulation.

At the beginning of the testing process, an oscilloscope tracked the frequency variations while an independent temperature sensor recorded the temperature changes. This setup was used because the temperature readings from the heat chamber were less accurate than those of the discrete temperature sensor. With this experimental setup, we acquired a comprehensive frequency-temperature relationship.

The experimental procedure began at an initial temperature of $-21^{\;\;\circ }$ C (this is the actual measured temperature we obtained when the heat chamber control was set to $-20^{\;\circ }$ C). From this baseline, the chamber temperature was increased in 2.5-degree increments. After each adjustment in the temperature chamber, a waiting period was allowed to ensure the chip temperature stabilized to the new setting. Stability was declared reached when the output frequency stabilized as the chip’s temperature reached equilibrium with the chamber temperature. Only after this stabilization of the output frequency were both frequency and temperature readings re-captured. This can take 4 min per point with our experimental setup. This sequence continued until the chamber temperature reached a high value of $85^{\;\circ }$ C. Following this, the process was reversed, with the chamber’s temperature decreasing in 2.5-degree steps. Once again, measurements were taken only after ensuring the stabilization of the output frequency, indicating the chip temperature’s alignment with the chamber temperature. This process continued until the system returned to $-21^{\;\circ }$ C.

To ensure consistency and repeatability of our findings, the entire process was replicated using a different chip under identical testbench conditions. The resulting data gave us a thorough understanding of the chip’s behavior and performance across a broad temperature range.

### 3.2. Evaluation Criteria

Computational tools and mathematical methodologies were employed to investigate the temperature-frequency relationship within the current-starved ROTS. Python scripting was utilized for data processing due to its efficiency and adaptability.

#### Sensitivity-Based Approach

The sensitivity-based approach was adopted to assess how small changes in temperature influence the frequency readings. The steps for this method are as follows:

- Polynomial Fit: To measure the error in the calibrated response, a 6th-degree polynomial was fitted to the measured data to represent as closely as possible the true temperature-frequency relationship.
(5) $$f\left(T\right)=a_{6}T^{6}+a_{5}T^{5}+a_{4}T^{4}+a_{3}T^{3}+a_{2}T^{2}+a_{1}T+a_{0}$$
  where f(T) represents the frequency as a function of temperature *T*, and $a_{0},a_{1},\ldots ,a_{6}$ are the polynomial coefficients obtained from the data.
- Sensitivity Calculation: The polynomial fitted to the measured data was differentiated to evaluate its sensitivity to temperature changes.
(6) $$s\left(T\right)=\frac{df(T)}{dT}$$
  where s(T) is the sensitivity of frequency to temperature at any given temperature *T*.
- Error Calculation: The error in frequency at each temperature point was calculated by comparing the fitted 6th-order polynomial’s value to the frequency values predicted by other approaches.
(7) $$\mathrm{frequency}\_\mathrm{error}\left(T\right)=f\left(T\right)-Y_{\mathrm{approach}}$$
- Temperature Error Using Sensitivity: The temperature error for the given frequency error was calculated using the polynomial sensitivity.
(8) $$\mathrm{temperature}\_\mathrm{error}\left(T\right)=\frac{\mathrm{frequency}\_\mathrm{error}(T)}{s(T)}$$
  Note that the temperature error becomes undefined if the sensitivity is zero at some temperatures.

The coefficient of determination was the primary metric to assess the fit between the suggested calibration techniques and the gathered data. It quantifies the degree to which the model can reproduce observed results, focusing on the percentage of the total outcome variability accounted for by the used model [27].

### 3.3. Fitting and Measurement Results

The calibration of the ring-oscillator-based temperature sensor introduces a distinct set of challenges, especially considering the sensor’s sensitivity to process variations during its fabrication. This section explores the trade-offs between accuracy and practicality. The assessment of the temperature sensor was made by applying the following steps:

- Three-point calibration: Using frequency measurements at −21 °C, 23 °C, and 81.5 °C.
- Two-point calibration: Using frequency measurements at −21 °C and 81.5 °C.
- Single-point calibration: Relying on a single frequency measurement at 23 °C.
- Linear Calibration: From frequency measurements at −21 °C and 81.5 °C.
- Second-Degree Polynomial Best Fit Approach: This best fit is made using all the recorded temperature-frequency data points (captured at 2.5 °C intervals) to interpolate the relationship.
- Uncalibrated Sensor: The typical process parameters were used to produce an uncalibrated nominal response based on post-layout circuit simulations.

**Figure 6:**
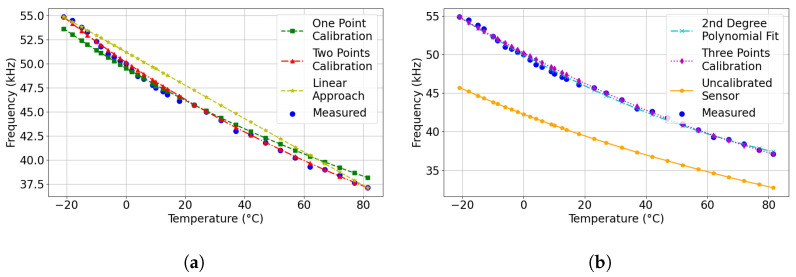
Performance evaluation of various calibration techniques: (**a**) Comparisons between One-Point Calibration, Two-Point Calibration, and Linear Calibration, and (**b**) Comparisons among Three-Point Calibration, 2nd Degree Polynomial Fit (Best-Fit), and the uncalibrated sensor.

**Figure 7:**
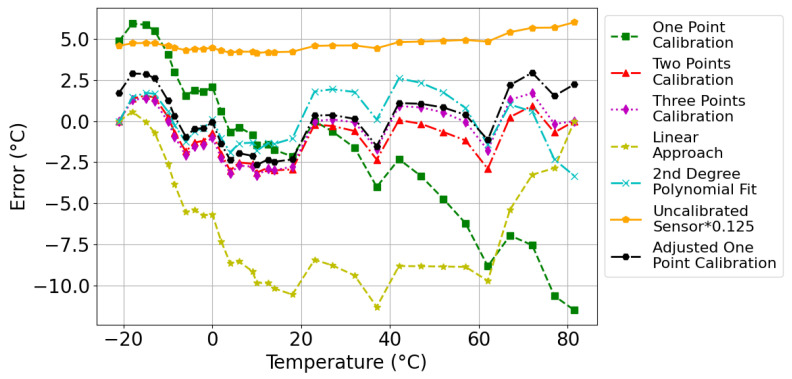
Temperature errors of different calibration techniques.

Several plots characterizing the temperature-frequency relationship generated with each approach were used to understand the calibration methods and their correlation with the measured data, as depicted in Figure 6. This graphical representation provides insight into the variance and alignment between the calibration techniques and the actual measurements. Based on this methodology, the resulting temperature errors for each technique at different temperature points were verified. These results are presented in Figure 7. Owing to the large temperature errors associated with the uncalibrated sensors, the displayed error results of the uncalibrated response were divided by eight to improve visualization clarity.

For statistical insight into the fit quality for each calibration method, each approach’s coefficient of determination ( $R^{2}$ values) is reported in Table 3.

As we delve into the results of our analysis, it is important to stress that our understanding of the Ring Oscillator (RO) calibration problem has evolved significantly over time, particularly after conducting the experiments that are reported here. A crucial starting point in this exploration is examining the performance of the uncalibrated sensor.

Our analysis revealed that the uncalibrated sensor, using typical process parameters, when simulated across varying temperatures, exhibited a negative $R^{2}$ value of −0.736, as shown in Table 3. Generally, $R^{2}$ values fall between 0 and 1, with negative values indicating that the model’s performance is significantly worse than a simple mean-based baseline. This usually occurs when a model does not include an intercept term, failing to capture the mean response within the dataset. The striking difference between the simulated results of the uncalibrated sensor and the measured frequencies, as illustrated in Figure 7, underscores the infeasibility of using uncalibrated ROs in scenarios where some level of accuracy is needed. This discovery was a crucial motivator for our investigation into various calibration methods, aiming to strike a balance between complexity and accuracy.

On the opposite end of the spectrum, we explored the second-degree polynomial approach, notable for its adaptability in capturing complex sensor behaviors using a polynomial equation. This method, while computationally demanding due to its reliance on a considerable number of data points—thirty-one points in our study—ensures high precision. The approach’s effectiveness is underscored by its impressive $R^{2}$ value of 0.9975, indicative of its remarkable accuracy. However, as Table 3 reveals, there are still temperature deviations that the model cannot fully account for, specifically a maximum positive deviation of 2.57 °C at 42 °C and a maximum negative deviation of −3.33 °C at 81.5 °C. This approach’s high $R^{2}$ value substantiates the quadratic nature of the temperature-frequency relationship. Recognizing that any quadratic polynomial can be defined with three distinct points led us to the Three-Point calibration method.

The Three-Point calibration method employs measurements at three different temperatures: −21 °C, 23 °C, and 81.5 °C. This method attained an $R^{2}$ value of 0.9956, confirming its high accuracy. The observed temperature deviations, as noted in Table 3, include a maximum positive deviation of +1.7 °C at 72 °C and a maximum negative deviation of −3.29 °C at 10 °C. While this method is precise, it requires experimental measurements at three distinct temperatures, which is a costly option still.

Our research aims to strike an optimal balance between simplicity and accuracy in temperature sensor calibration. To this end, we developed the Two-Point calibration method, requiring just two temperature reference points at $-21^{\;\circ }C$ and $81.5^{\;\circ }C$ . This approach demonstrates exceptional precision, as indicated by its $R^{2}$ value of 0.9958.

Comparing this with previous studies, our Two-Point calibration method showcases competitive accuracy, even outperforming some traditional methods that rely on circuit trimming. For instance, in the study by [11], temperature deviations of $3.00^{\;\circ }C$ at $100^{\;\circ }C$ and $-3.00^{\;\circ }C$ at $20^{\;\circ }C$ were reported. Similarly, the study in [12] observed deviations of $2.60^{\;\circ }C$ at $80^{\;\circ }C$ and $-3.40^{\;\circ }C$ at $60^{\;\circ }C$ . These deviations are slightly higher than those we observed using our Two-Point calibration method, which achieved maximum positive and negative deviations of $+1.56^{\;\circ }C$ at $-15^{\;\circ }C$ and $-3.13^{\;\circ }C$ at $+10^{\;\circ }C$ , respectively.

Our results are particularly noteworthy given that our method does not require the complex circuit adjustments often employed in other studies. This simplification not only makes the calibration process more straightforward but also broadens the potential for its application in various semiconductor contexts where circuit trimming may be impractical or unavailable.

The One-Point Calibration Method, while offering ease of calibration at ambient conditions without a temperature chamber, presents certain limitations in terms of accuracy. This method, which leverages a single data point for calibration, achieved an $R^{2}$ value of 0.9808, as reported in Table 3. Temperature deviations observed with this simple method reach $+5.94^{\;\circ }C$ at $-18^{\;\circ }C$ and $-11.5^{\;\circ }C$ at $81.5^{\;\circ }C$ , highlighting the inherent inaccuracies of this approach.

While this method proved to be fairly accurate around the calibration temperature of $23^{\;\circ }C$ , significantly larger temperature errors were observed at other points. It is worth noting that the minor residual error at $23^{\;\circ }C$ was primarily due to the use of a sixth-degree polynomial for representing the measured data, which led to a slight discrepancy at this specific point.

During the evaluation of the One-Point calibration method, it was noted that while the approach was fundamentally sound, it exhibited a consistent linear residual error. To address this, we refined the calibration strategy by implementing a first-order linear correction to the existing quadratic calibration curve. This corrective step was crucial in enhancing the method’s precision.

In the process of developing this refined strategy, we conducted analyses on two separate temperature sensor chips. Interestingly, each chip yielded a linear correction equation that was remarkably similar to the other. This similarity indicated a consistent pattern in the calibration error across different chips. Despite the close alignment of these linear equations, we decided to average the linear correction equations from both chips. This averaging resulted in a unified correction formula that was applicable universally, rather than being specific to a single chip.

Applying this averaged linear correction to the One-Point calibration significantly improved the accuracy of the temperature measurements. The residual error was reduced to within $\pm 2^{\;\circ }C$ , a marked improvement from the initial uncorrected One-Point method. This enhancement in accuracy highlights the effectiveness of the Corrected One-Point Calibration method. Not only does it demonstrate the method’s robustness across different chips, but it also validates its ability to provide accurate temperature readings over a broad range of temperatures.

Finally, the simple Linear Approach simplifies the calibration process by relying on a straight linear relationship between two data points. As per Table 3, it only attains an $R^{2}$ value of 0.9287. In this case, however, simplicity comes at the price of large temperature deviations: 6.72 °C at 18 °C and −0.28 °C at −18 °C.

As we approach the conclusion of this research, it is essential to investigate the practical implications of our findings, particularly regarding their potential application. Our proposed design, although demonstrated in a software simulation, is intended for future adaptation to hardware platforms like Field-Programmable Gate Arrays (FPGAs). This is particularly relevant in industrial settings involving system-on-chip architectures, where real-time processing and swift data handling are paramount.

The practical use of our fine-tuning calibration technique merits consideration, particularly regarding its implementation, while considering its influence on sensor precision and adaptability to diverse manufacturing processes. Key to its deployment will be its performance under real-world conditions, its scalability for mass production, and its compatibility with existing designs. These aspects are crucial in assessing the real-world viability and applicability of the calibration technique.

However, it is important to note that our calibration approach necessitates additional computations following each temperature measurement. This requirement contrasts with traditional trimming methods that usually rely on simpler counting mechanisms. The complexity of the computations needed for accurate temperature-to-frequency mapping could demand substantial processing power. Thus, unless an embedded processor is available as in many modern FPGAs, practical hardware implementation may require extra chip area, enabling real-time processing while maintaining the accuracy that we have demonstrated in this study. The availability of suitable computing resources is a key consideration that allows for simplifying sensor complexity, thus enabling practical temperature sensing and system monitoring, striking a balance between accuracy and efficiency.

In summary, while the present paper and the cited works leverage the principle of Two-Point calibration, the methodologies, circuits, and applications using it are quite different. The calibration approach proposed in this paper showcases that even without resorting to circuit trimming, one can achieve very high accuracy.

## 4. Conclusions

In this paper, we presented a comprehensive study on the calibration of an uncalibrated ring-oscillator-based temperature sensor, developed using the 0.18- μ m SOI XFAB process. The sensor is primarily designed for efficient thermal monitoring in system-on-chip (SoC)-based DC-DC converters.

Our research focused on exploring and characterizing a range of trimming-free calibration techniques, spanning from zero to thirty-one frequency-temperature measurement points. The key contribution of our work lies in the successful implementation of these calibration techniques, especially the Corrected One-Point calibration method. Remarkably, this method demonstrated a very favorable trade-off between simplicity and accuracy as it uses a single ambient temperature measurement for calibration, thereby eliminating the need for temperature chamber testing. This novel approach significantly simplifies the calibration process, making it both practical and cost-effective. After an adjustment involving a first-order polynomial correction, the One-Point Calibration method successfully reduced the maximum error to within ±2.95 °C, highlighting its potential for applications requiring a balance between simplicity and accuracy.

Additionally, the Two-Point calibration method demonstrated improved precision with minimal deviation, achieving a maximum positive error of +1.56 °C at −15 °C and a maximum negative error of −3.13 °C at +10 °C, accompanied by an impressive $R^{2}$ value of 0.9958. Equally effective, the Three-Point calibration method achieved a comparable $R^{2}$ value of 0.9956, underscoring that both methods deliver similar levels of accuracy. This parity in performance validates the efficacy of these calibration strategies across various contexts, offering flexible options for temperature sensor calibration without compromising precision.

Our findings illustrate that it is feasible to achieve competitive results in temperature sensor calibration without resorting to circuit trimming. This not only offers a viable alternative to conventional calibration methods but also serves as a complementary approach, particularly in scenarios where trimming is not feasible or desired. The results of our study, particularly the effective use of the Corrected One-Point calibration method, underscore the inherent trade-off between complexity and accuracy in sensor calibration and demonstrate the potential of our methods in addressing this challenge.

As a future direction, transitioning the calibration algorithm from its current software-based simulation to an FPGA interface is anticipated. This shift is expected to unlock the potential for faster processing speeds and real-time application, aligning with the growing demand for efficient temperature monitoring in complex industrial systems.

## Figures and Tables

**Table 1:** Oscillator-based temperature sensors in the literature.

Reference	Ring Oscillator Topology	Calibration Method	Accuracy
[11]	two ring oscillators and a subtractor	one trimming point	[−2.9 °C, 2.7 °C]
[12]	ring oscillator, biased by PTAT, sink resistor	two trimming points	±3 °C
[13]	RS register	resistor trimming, 5-bit	±1 °C
[14]	current-controlled	N/A	0.7 °C / bit
[15]	current-starved, digital control	output capacitor trimming, 4-bit	0.4 °C / bit
[16]	current-starved, digital control	N/A	0.18 °C / bit
[17]	delay line	digital comparator and time amplifier	±0.7 °C
[18]	parallel ring oscillators	zero temperature coefficient ring oscillator and voltage mapping	[−1.76 °C, 1.96 °C]
[19]	current-controlled oscillator	resistor trimming, two points	±1.5 °C
[20]	two ring oscillators, bandgap concept	two trimming points	[−1.7 °C, 2.1 °C]

**Table 2:** Oscillator-based Temperature Sensor Transistor Dimensions.

Transistor Name	Total Width	Total Length	Transistor Name	Total Width	Total Length
Q1	220 nm	10 μ m	Q4	220 nm	2 μ m
Q2	1.1 μ m	2 μ m	P1,x	440 nm	2 μ m
Q3	440 nm	2 μ m	P2,x	20 μ m	10 μ m
N1,x	220 nm	2 μ m	B1	2 μ m	500 nm
N2,x	10 μ m	10 μ m	B2	1 μ m	500 nm
B3	8 μ m	500 nm	B4	4 μ m	500 n

The variable ‘x’ represents the stage number, which is an integer number ranging from 1 to 9.

**Table 3:** Comparative analysis of calibration methods: Coefficient of determination ( $R^{2}$ ) and temperature errors.

References	Method	CMOS Technology	$R^{2}$	Max Positive Error (°C)|Corresponding Temp. (°C)	Max Negative Error (°C)|Corresponding Temp. (°C)
This Work	Uncalibrated	Xfab180 nm	−0.736	+48.12|+81.5	N/A|N/A
This Work	2nd Degree Polynomial Fit	Xfab180 nm	0.9975	+2.59|+42	−3.33|+81.5
This Work	3-Point Calibration	Xfab180 nm	0.9956	+1.70|+72	−3.29|+10
This Work	2-Point Calibration	Xfab180 nm	0.9958	+1.56|−15	−3.13|+10
This Work	Linear Fit	Xfab180 nm	0.9364	+0.57|−18	−11.30|+37
This Work	1-Point Calibration	Xfab180 nm	0.9808	+5.94|−18	−11.50|+81.5
This Work	Corrected 1-Point Calibration	Xfab180 nm	0.9957	+2.95|+72	−2.66|+10
[11]	Ring oscillator: Trimming 1-point	TSCM65 nm	N/A	+3.00|100	−3.00|+20
[12]	Ring oscillator: Trimming 2-point	TSCM65 nm	N/A	+2.60|+80	−3.40|+60
[18]	ROTS: Trimming 2-point	TSMC180 nm	N/A	+2.00|−25	−1.60|+25
[17]	Delay Line: Trimming 2-point	TSCM65 nm	N/A	+1.00|0	−0.80|+35

## Data Availability

Data are contained within the article.
